# The Role of T Cells Reactive to the Cathelicidin Antimicrobial Peptide LL-37 in Acute Coronary Syndrome and Plaque Calcification

**DOI:** 10.3389/fimmu.2020.575577

**Published:** 2020-10-06

**Authors:** Fernando Chernomordik, Bojan Cercek, Wai Man Lio, Peter M. Mihailovic, Juliana Yano, Romana Herscovici, Xiaoning Zhao, Jianchang Zhou, Kuang-Yuh Chyu, Prediman K. Shah, Paul C. Dimayuga

**Affiliations:** Oppenheimer Atherosclerosis Research Center, Department of Cardiology, Smidt Heart Institute, Cedars-Sinai Medical Center, Los Angeles, CA, United States

**Keywords:** acute coronary syndrome, T cells, atherosclerosis, cathelicidin, LL-37, mCRAMP

## Abstract

The human cationic anti-microbial peptide LL-37 is a T cell self-antigen in patients with psoriasis, who have increased risk of cardiovascular events. However, the role of LL-37 as a T cell self-antigen in the context of atherosclerosis remains unclear. The objective of this study was to test for the presence of T cells reactive to LL-37 in patients with acute coronary syndrome (ACS). Furthermore, the role of T cells reactive to LL-37 in atherosclerosis was assessed using apoE−/− mice immunized with the LL-37 mouse ortholog, mCRAMP. Peripheral blood mononuclear cells (PBMCs) from patients with ACS were stimulated with LL-37. PBMCs from stable coronary artery disease (CAD) patients or self-reported subjects served as controls. T cell memory responses were analyzed with flow cytometry. Stimulation of PBMCs with LL-37 reduced CD8+ effector T cell responses in controls and patients with stable CAD but not in ACS and was associated with reduced programmed cell death protein 1 (PDCD1) mRNA expression. For the mouse studies, donor apoE−/− mice were immunized with mCRAMP or adjuvant as controls, then T cells were isolated and adoptively transferred into recipient apoE−/− mice fed a Western diet. Recipient mice were euthanized after 5 weeks. Whole aortas and hearts were collected for analysis of atherosclerotic plaques. Spleens were collected for flow cytometric and mRNA expression analysis. Adoptive transfer experiments in apoE−/− mice showed a 28% reduction in aortic plaque area in mCRAMP T cell recipient mice (P < 0.05). Fifty six percent of adjuvant T cell recipient mice showed calcification in atherosclerotic plaques, compared to none in the mCRAMP T cell recipient mice (Fisher’s exact test *P* = 0.003). Recipients of T cells from mice immunized with mCRAMP had increased IL-10 and IFN-**γ** expression in CD8+ T cells compared to controls. In conclusion, the persistence of CD8+ effector T cell response in PBMCs from patients with ACS stimulated with LL-37 suggests that LL-37-reactive T cells may be involved in the acute event. Furthermore, studies in apoE−/− mice suggest that T cells reactive to mCRAMP are functionally active in atherosclerosis and may be involved in modulating plaque calcification.

## Introduction

Adaptive immunity has a major role in atherosclerosis (1), the underlying cause of coronary artery disease (CAD), associated with a variety of antigens that have been described (2). However, other potential self-antigens remain unknown (3). It has been proposed that in the chronic inflammatory state present in atherosclerosis, tolerance to self-antigens could be broken through several potential mechanisms, implicating an autoimmune component in atherosclerosis (4). This is supported by the reported correlation between atherosclerosis and T Effector Memory cells (5). Furthermore, T Effector Memory cell density is associated with atherosclerotic plaque stage in humans (6). Rupture of the atherosclerotic plaque is the most common cause of acute coronary syndromes (ACS).

Antimicrobial peptides are an important part of the innate immune system and they are active against pathogens directly through their antimicrobial properties, or through their immunomodulatory effects (7). The human antimicrobial peptide LL-37, a cleavage product of the cathelicidin proprotein hCAP-18, is present in human atherosclerotic plaques (8), and is associated with platelet activation and induction of thrombosis (9), with higher serum levels in coronary circulation compared to systemic levels in patients with ST elevation myocardial infarction (STEMI) (10). Moreover, LL-37 is present in neutrophil extracellular traps (NETs), which have been implicated in atherogenesis (11). They are associated with self-immunity as self-DNA/LL-37 complexes that aggravate atherogenesis (12). Interestingly, NET burden was associated with infarct size and negatively associated with ST segment resolution in patients with STEMI (13). Furthermore, LL-37 induced differentiation of human mononuclear cells into bone-forming cells (14), suggesting a potential role in calcific mineralization. LL-37 is a T cell self-antigen in patients with psoriasis (15), who have increased risk of cardiovascular morbidity and mortality (16, 17). It is not known if there is a self-reactive T cell response to LL-37 as a self-antigen in the context of atherosclerosis and if so, whether it is beneficial or pathogenic.

**FIGURE 1:**
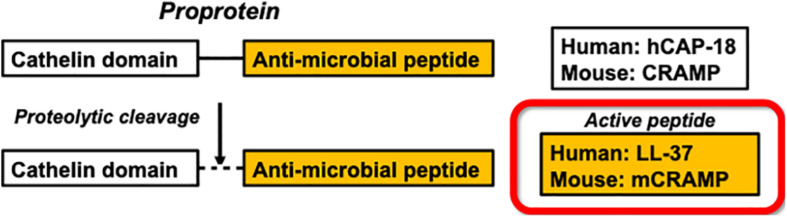
Enzymatic processing of the human antimicrobial peptide LL-37 and the mouse ortholog mCRAMP.

CRAMP is the mouse homolog of the human cathelicidin proprotein hCAP-18, which like CRAMP, is proteolytically cleaved to generate a cathelin-like domain and the cationic anti-microbial domain LL-37, or mCRAMP in mice (Figure 1). Similar to LL-37 in humans, the proprotein CRAMP has been linked to atherogenesis in apoE−/− mice as suggested by experiments showing that Cramp−/− apoE−/− mice have less atherosclerosis compared to apoE−/− mice (18). Our group has previously shown that immunization with a low dose of the murine proprotein CRAMP was associated with decreased atherosclerosis in apoE−/− mice (19).

The objective of this study was to test the role of LL-37 as a potential T cell self-antigen in patients with ACS. Furthermore, the role of T cells reactive to LL-37 in atherosclerosis was assessed using apoE−/− mice immunized with the LL-37 mouse ortholog, mCRAMP.

## Materials and Methods

### Human PBMC

The protocols were approved by the Cedars-Sinai Institutional Review Board (IRB). Peripheral blood mononuclear cells (PBMCs) were isolated from blood collected from 10 patients with ACS within 72 h of admission to the Cedars-Sinai Cardiac Intensive Care Unit. Patients were consented under the approved IRB protocol Pro48880. Exclusions were inability to give informed consent, age less than 18 years old, active cancer treated with chemotherapy or radiation, patients taking immune-suppressive drugs, and pregnant women. PBMCs from 10 stable CAD patients were isolated from blood collected on the day of coronary angiography, consented under the approved IRB protocol Pro50839 with the same exclusions. Consented data use was limited to age, sex, LDL levels, and use/non-use of cholesterol-lowering medication. PBMCs were isolated using Ficoll density gradient centrifugation and cryo-preserved in commercially available cryogenic solution (Immunospot) in liquid nitrogen. Cryo-preserved PBMCs from self-reported controls (*N* = 15) were purchased from a commercial source (Immunospot).

### Peptide Stimulation of Human PBMC

Cryo-preserved PBMCs were thawed, rinsed in anti-aggregation solution (Immunospot), and seeded in culture plates at a density of 3 × 10^6^cells per ml of RPMI 1640 medium supplemented with 10% heat-inactivated pooled human serum and 1× antibiotic/antimycotic. Peptides (LifeTein) used for stimulation corresponded to LL-37 and the truncated cathelin domain of hCAP-18 [cat-hCAP-18 (aa 39-136)]. Cells were stimulated with one of the following: 20 μg/ml LL-37 or cat-hCAP-18 peptide, 0.5× T cell stimulation cocktail containing PMA and ionomycin (Thermo Fisher). Culture medium was added at ^1^/_3_ of the starting volume 48 h later to replenish the nutrients in the medium. Cells were harvested 72 h after seeding, stained for viability (LIVE/DEAD Fixable Aqua Dead Stain Kit, Thermo Fisher), and subjected to cell surface staining for flow cytometry using the following antibodies: CD3, CD4, CD8, CD45RA, CD45RO, CD62L, and CD197 (CCR7). Isotypes were used as staining control. CD4+ or CD8+ T Effector cells were gated on CD45RO+CD62L(−)CD197(−). T Effector Memory cells were CD45RO+CD45RA(−) CD62L(−)CD197(−), T Effector Memory RA+ cells were CD45RO+CD45RA(+)CD62L(−)CD197(−). Results were tabulated as Response Index using the following calculation (20):

$$\frac{\left(\%peptidestimulation-\%nostimulation\right)}{\left(\%cocktailstimulation\right)}\times 100$$

The results are expressed as Response Index to account for inherent variations introduced by culturing cells in vitro over time, controlled for by assessing response relative to baseline cell phenotype (% no stimulation) and maximal stimulation (% cocktail stimulation) for each subject PBMC. Each data point represents one subject.

### PDCD1 mRNA Expression

In samples with sufficient cell numbers, stimulated PBMCs cultured for 72 h were collected for RNA extraction using Trizol (Thermo Fisher) and subjected to qRT-PCR with SYBR green and a primer pair for human PDCD1. Cyclophilin A served as the reference gene. Results were expressed as fold-change relative to non-treated cells of each sample using the Ct_ΔΔ_ method.

### IFN-**γ** ELISA

Conditioned medium was collected after 72 h of LL-37 stimulation of PBMCs and IFN-**γ** was measured using a commercially available ELISA kit (Abcam) according to manufacturer’s instructions.

### MHC Blocking

PBMCs from control samples were stimulated with LL-37 alone or in the presence of either anti-human HLA-A, B, C (clone W632) or anti-human HLA-DR (clone L243) monoclonal antibody (Biolegend; 30 μg/ml) for 72 h and stained for flow cytometry as described above.

### Animal Experiments

The studies were approved by the Cedars-Sinai Institutional Animal Care and Use Committee. Male apoE−/−mice were purchased from Jackson Laboratory at 6 weeks of age and housed in a specific pathogen-free facility, kept on a 12-h day/night cycle, and had unrestricted access to water and food.

### mCRAMP Immunization

The mCRAMP peptide was purchased (AnaSpec) with >95% purity according to the manufacturer. Donor mice were subcutaneously immunized with either 100 μg mCRAMP in adjuvant [Adju-Phos (Brenntag, 12.5 μl of a 2% solution) and monophosphoryl lipid A (MPLA-SM VacciGrade, InvivoGen, 10 μg)] or adjuvant alone in a final volume of 200 μl using PBS as vehicle. One group of mice was fed normal chow and immunized at 7, 10, and 12 weeks of age. Mice were then euthanized and splenocytes isolated to assess T cell response at 13 weeks of age using flow cytometry. Another group of mice was fed normal chow and immunized at 7, 10, and 12 weeks of age. The mice were then euthanized as T cell donors at 13 weeks of age. Recipient mice were fed high fat diet consisting of 21% fat, 0.15% cholesterol (TD.88137, Envigo) starting at 7 weeks of age until euthanasia at 23 weeks of age.

### Isolation and Transfer of T Cells

After collection of donor mouse splenocytes, red blood cells were lysed using RBC lysis buffer (BioLegend), washed with PBS, and cells from the same group were pooled. Splenocytes were enriched for T cells using a commercially available mouse T cell magnetic isolation kit (ThermoFisher) according to the manufacturer’s protocol. T cells were enriched to ∼90% assessed by flow cytometry. After counting the isolated T cells, a suspension of 2 x10^6^ T cells in PBS was injected by tail vein injection into 18 week-old recipient mice fed with high fat diet for 11 weeks. High fat diet feeding in recipient mice continued for an additional 5 weeks and mice were euthanized at 23 weeks of age.

### Tissue Harvesting

At 23 weeks of age, recipient mice were euthanized and spleens, aortas and hearts were collected. Serum was collected for cholesterol level measurement. Spleens were collected for flow cytometric staining and analysis. A small portion of the spleen was used for RNA extraction. The aortas were dissected free of connective tissue and fat, and stained with Oil Red O for en face lipid staining (19). Aortic size and plaque content were quantified by a blinded observer using image analysis software (ImagePro Plus version 4.0, Media Cybernetics Inc., Rockville, Maryland). Heart bases were imbedded in OCT (Optimum Cutting Temperature, Tissue-Tek) and frozen for cryo-sectioning. Ten-micron cryosections of the aortic sinus were collected. Lipid was stained using Oil-Red-O. Staining for macrophage was performed using CD68 antibody. Collagen area was assessed using Masson trichrome stain. Three slides of approximately 0.1-millimeter intervals for each animal were used for each stain and averaged. Image analysis was performed using ImagePro. Tissue calcification in aortic sinus plaques was confirmed by Alizarin Red-S stain.

### Flow Cytometry

Splenocytes were stained for viability and surface markers CD3, CD4, CD8, CD44, and CD62L. Intracellular staining for FoxP3 was performed after surface marker staining followed by fixation and permeabilization (eBioscience). For intracellular cytokine staining, cells were resuspended in 10% heat inactivated FBS-RPMI medium containing 1× Monensin (Invitrogen) and cultured at 37°C and 5% CO_2_ for 4 h. After viability and surface marker staining, cell fixation and permeabilization was performed followed by intracellular staining for IL-10 and IFN-γ for flow cytometry.

### CD107a

For degranulation assay to measure CD8+ T cell cytolytic activity, splenocytes were incubated with 2.5 μg/ml fluorescent conjugated CD107a in RPMI for 1 h followed by the addition of Monensin and incubation for another 4 h. Cells were collected and stained for surface markers for flow cytometry.

### Splenic mRNA Expression

Splenic RNA was isolated using Trizol and subjected to qRT-PCR using primer pairs for mouse IL-1β, Pdcd1, Ctla4, Wnt10b, Runx2, RANKL, and osteocalcin. GAPDH was used as reference gene. Data were analyzed using the Ct_ΔΔ_ method with one Adjuvant sample as calibrator. Results are expressed as fold change relative to Adjuvant.

### ELISA

Mouse serum levels of soluble RANKL and undercarboxylated osteocalcin, the active form of osteocalcin, were measured using commercially available ELISA kits (ThermoFisher and MyBioSource, respectively) according to manufacturers’ instructions.

### Statistical Analysis

Statistical analysis was performed using R software version 3.5 (R Core Team, 2018) and GraphPad Prism version 7 (GraphPad Software, La Jolla, California). Data are presented as mean ± standard deviation. For multiple group analysis, significance for normally distributed samples was tested using ANOVA followed by Holm-Sidak’s multiple comparisons test. Significance for non-normally distributed samples was tested using Kruskal-Wallis test followed by Dunn’s multiple comparisons test. Correlation was analyzed using the Spearman test with a two-tailed *P*-value. For two-group analysis, following normality testing, differences between groups were performed using *t*-test or Wilcoxon test, accordingly. A *P*-value < 0.05 was considered significant, but data trends were also noted.

## Results

### T Cell Immune-Reactivity to the Human Cationic Antimicrobial Peptide LL-37 in Acute Coronary Syndrome Patients

**FIGURE 2:**
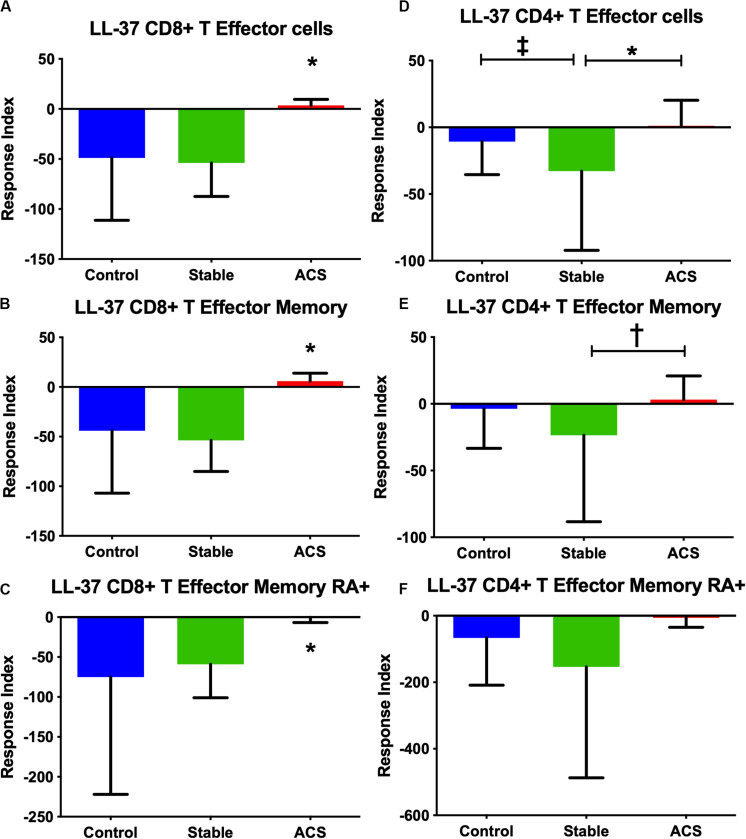
PBMC T Effector cell response to stimulation with the human antimicrobial peptide LL-37. CD8+ **(A–C)** and CD4+ **(D–F)** Memory T cell responses to stimulation of peripheral blood mononuclear cells from self-reported controls (Control), stable coronary artery disease (Stable), and acute coronary syndrome (ACS) patients. T Effector Memory **(B,E)** and T Effector Memory RA+ **(C,F)** were based on CD45RO/CD45RA flow cytometric stain as detailed in the gating scheme described in Supplementary Figure 1. Control *N* = 15; Stable N = 10; ACS *N* = 10. *P < 0.05 ACS vs Control or Stable CAD **(A–C)** and Stable vs ACS **(D)**; ^‡^*P* = 0.053 Control vs Stable; ^†^*P* = 0.055 Stable vs ACS. Kruskal-Wallis and Dunn’s multiple comparisons test.

**FIGURE 3:**
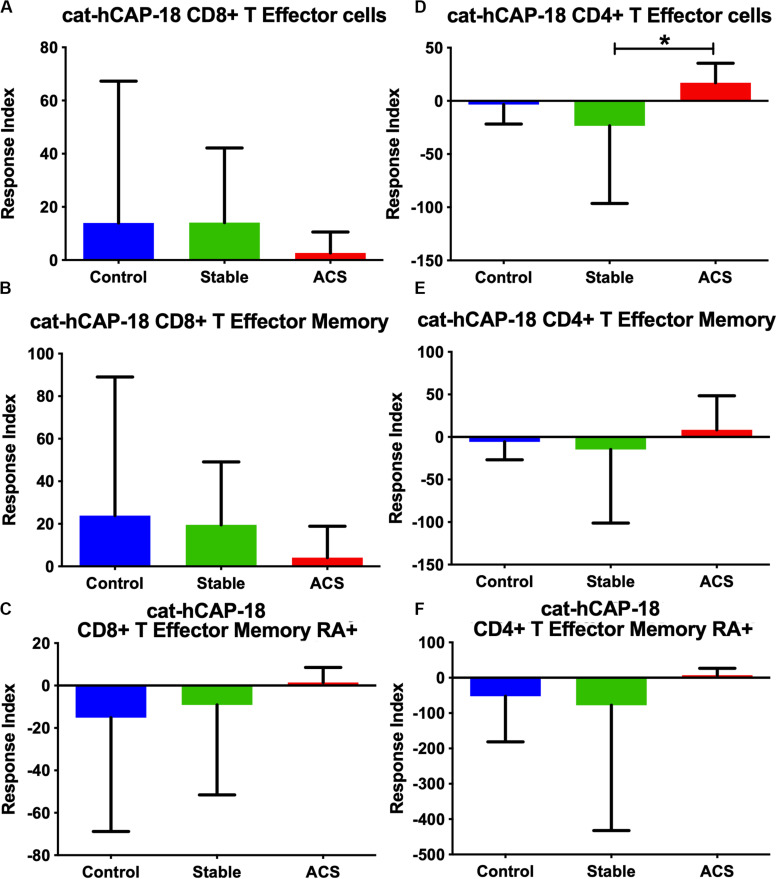
PBMC T Effector cell response to stimulation with the cathelin domain of the human proprotein hCAP-18, cat-hCAP-18. CD8+ **(A–C)** and CD4+ **(D–F)** Memory T cell responses to stimulation of peripheral blood mononuclear cells from self-reported controls (Control), stable coronary artery disease (Stable), and acute coronary syndrome patients (ACS). T Effector Memory **(B,E)** and T Effector Memory RA+ **(C,F)** were based on CD45RO/CD45RA flow cytometric stain as detailed in the gating scheme described in Supplementary Figure 1. Control *N* = 15; Stable CAD *N* = 10; ACS *N* = 9; *P < 0.05 Stable vs ACS. Kruskal-Wallis and Dunn’s multiple comparisons test.

**FIGURE 4:**
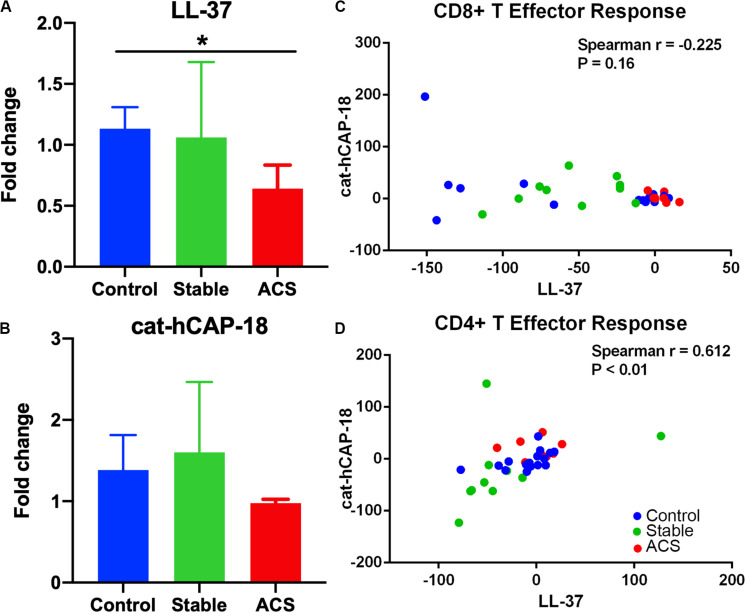
Immune checkpoint PDCD1 mRNA expression and correlation in T Effector response to LL-37 and cat-hCAP-18. Programmed cell death protein 1 (PDCD1) mRNA expression in peripheral blood mononuclear cells stimulated with the human antimicrobial peptide LL-37 **(A)** or the cathelin domain of the proprotein hCAP-18, cat-hCAP-18 **(B)**. Control *N* = 6; Stable = 5; ACS = 5. *P < 0.05 (ACS vs Control; Kruskal-Wallis and Dunn’s multiple comparisons test). Correlation plot between CD8+ T Effector response to LL-37 and cat-hCAP-18 **(C)**. Correlation plot between CD4+ T Effector response to LL-37 and cat-hCAP-18 **(D)**.

Given the role of anti-microbial peptides as potential self-antigens in atherosclerosis, and the possible association with acute events, we tested if the cleaved fragment of hCAP-18, the cationic antimicrobial peptide LL-37, would induce differential T cell immune responses in patients with ACS. Peripheral blood mononuclear cells (PBMCs) from self-reported healthy controls (Controls), patients with stable CAD (Stable) or ACS were stimulated with LL-37 for 72 h. PBMCs stimulated with the cathelin domain of hCAP-18 (cat-hCAP-18) served as control. Baseline characteristics of the subjects are detailed in Table 1. Stimulation with LL-37 resulted in reduced CD8+ Effector T cell response, in both T Effector Memory (TEM) and T Effector Memory RA+ (TEMRA) cells in PBMCs from controls and patients with stable CAD, while PBMCs from patients with ACS were resistant to this reduction (Supplementary Figure 1 and Figures 2A–C). CD4+ Effector T cell responses were trending similar to CD8+ Effector T cells (Figures 2D–F). There was no significant difference in CD8+ Effector T cell response when cells were stimulated with cat-hCAP-18 (Figure 3). There was reduced expression of programmed cell death protein 1 (PDCD1) mRNA in PBMCs from patients with ACS stimulated with LL-37 compared to PBMCs from controls (Figure 4A), but no difference was noted between groups in PDCD1 mRNA expression when cells were stimulated with cat-hCAP-18 (Figure 4B).

**TABLE 1:** Characteristics of human subjects.

	Control (*N* = 15)	Stable CAD (*N* = 10)	ACS (*N* = 10)
Mean age	58.7 ± 10.2	75.6 ± 9.0	59.3 ± 16.4
Male sex	70%	80%	70%
Mean LDL cholesterol (mg/dL)	N/A	86.62 ± 40.22*	105.96 ± 44.85^†^
Use of cholesterol-lowering medications on admission	N/A	90%	40%

ACS: acute coronary syndrome; CAD: coronary artery disease; LDL: low density lipoprotein; N/A: not available. *Based on chart review. ^†^At enrollment.

The nature of the self-reactive responses to cat-hCAP-18 and LL-37 were investigated further by testing the relationship between the T cell response to both antigens in each subject. There was significant correlation in CD4+ Effector T cell response (Figure 4D) to cat-hCAP-18 and LL-37, but not in CD8+ Effector response (Figure 4C). The results suggest that there is potentially shared antigenic reactivity to cat-hCAP-18 and LL-37 in CD4+ T cell responses. IFN-γ measured in conditioned medium from PBMCs stimulated with LL-37 showed qualitative difference in ACS compared to control and stable CAD patients (Supplementary Figure 2). The results suggest that LL-37 reactive T cells may be involved in the acute event. Additionally, Effector T cell responses in control PBMCs stimulated with LL-37 was partially reversed by blocking with HLA Class-I antibody (Supplementary Figure 3) suggesting HLA Class-I mediated response.

The T cell response in ACS patients was further subdivided into patients with their first ACS event and those who had a recurrent event. The CD8+ T Effector response to LL-37 was consistent between first and recurrent ACS patients, compared to patients with Stable CAD (Supplementary Figures 4A–C). On the other hand, CD4+ T Effector response was significantly higher only in the recurrent ACS patients but not in the first ACS event compared to Stable patients (Supplementary Figure 4D). This suggests that CD8+ T cell response to LL-37 persists in immunologic memory and is a common response in both first event ACS and recurrent ACS. CD4+ T cell Effector response on the other hand seems to have evolved and is more prominent in the recurrent ACS. No significant differences were observed in cat-hCAP-18 response between first ACS event and recurrent ACS patients in CD8+ T Effector (Supplementary Figures 5A–C) and CD4+ T Effector (Supplementary Figures 5D–F) responses.

To investigate the potential role of T cells that are self-reactive to the cationic antimicrobial peptide in atherosclerosis, we used the apoE−/− mouse model of atherosclerosis and immunization with the mouse ortholog of LL-37 called mCRAMP.

### Immunization Provokes a Self-Reactive T Cell Response to mCRAMP in apoE−/− Mice

**FIGURE 5:**
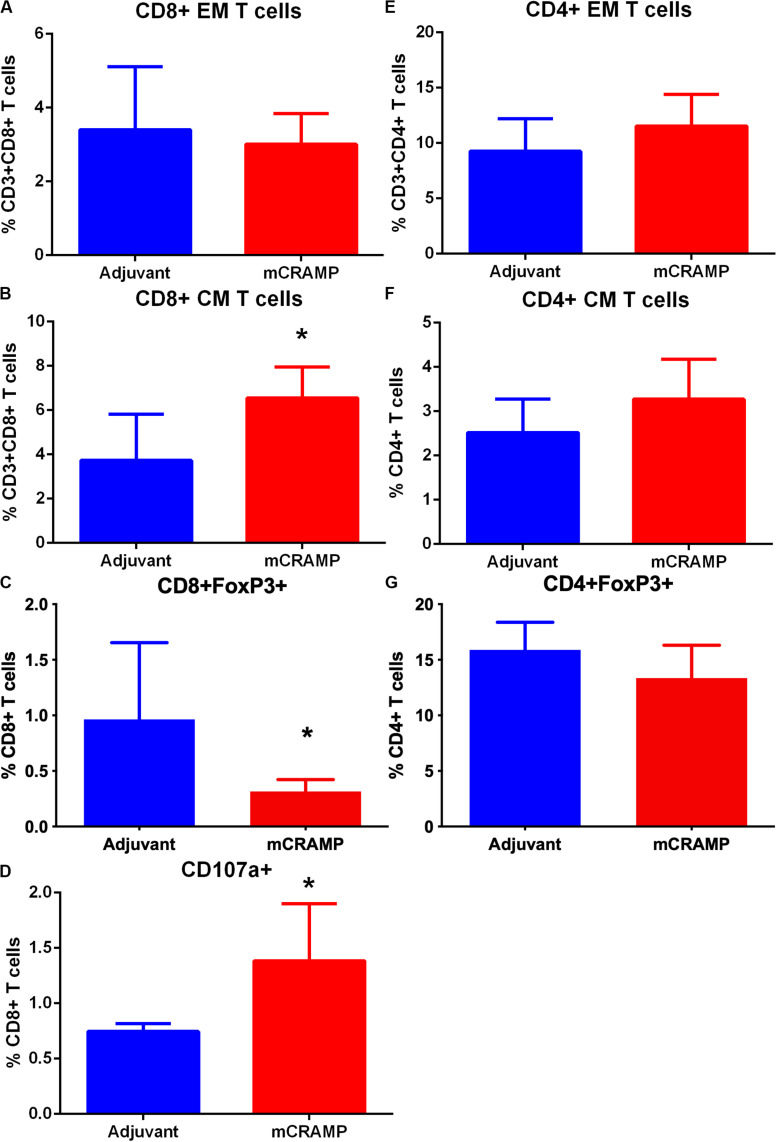
Memory T cell response of splenocytes to immunization of apoE–/– mice with the murine antimicrobial peptide mCRAMP. CD8+ Effector Memory (EM) T cells **(A)**, Central Memory (CM) T cells **(B)**, CD8+FoxP3+ T cells **(C)** and CD8+CD107a+ T cells **(D)** in mice immunized with mCRAMP compared to adjuvant. *N* = 5 each. CD4+EM T cells **(E)**, CD4+ CM T cells **(F)**, and CD4+FoxP3+ T cells **(G)** in mice immunized with mCRAMP compared to Adjuvant. *N* = 5 each; *P < 0.05, *t*-test. Gating scheme for memory T cells and FoxP3+ T cells shown in Supplementary Figure 6. Gating scheme for CD8+CD107a+ T cells shown in Supplementary Figure 7.

To investigate the potential role of T cells reactive to the mouse cationic antimicrobial peptide mCRAMP in the male apoE−/− mouse model of atherosclerosis, we first tested if T cells were reactive to mCRAMP. ApoE−/− mice fed with normal chow were immunized with mCRAMP at 7, 10, and 12 weeks of age and euthanized 1 week later for assessment of T cell response. Mice injected with adjuvant alone served as control. There was no difference in CD8+ Effector Memory T cells (Supplementary Figure 6 and Figure 5A) but a significant increase in CD8+ Central Memory (CM) T cells (Figure 5B) in splenocytes from apoE−/− mice immunized with mCRAMP. Additionally, there was decreased CD8+FoxP3+ cells (Supplementary Figure 6 and Figure 5C) and increased cytolytic activity, assessed by CD8+CD107a+ T cells (Supplementary Figure 7 and Figure 5D), in splenocytes of mice immunized with mCRAMP compared to adjuvant. No differences were observed in CD4+ memory T cell subsets or in CD4+FoxP3+ Treg cells (Figures 5E–G).

### mCRAMP-Primed T Cells in Atherosclerosis

To assess whether mCRAMP-primed T cells are functionally involved in modifying atherosclerosis, adoptive transfer of donor T cells from apoE−/− mice immunized with mCRAMP or adjuvant alone was performed on apoE−/− recipient mice that had been fed high fat diet for 11 weeks prior to transfer. The 11-week feeding with high fat diet assured that the recipient mice were already primed for atherosclerosis. Recipient mice were euthanized 5 weeks after cell transfer.

**FIGURE 6:**
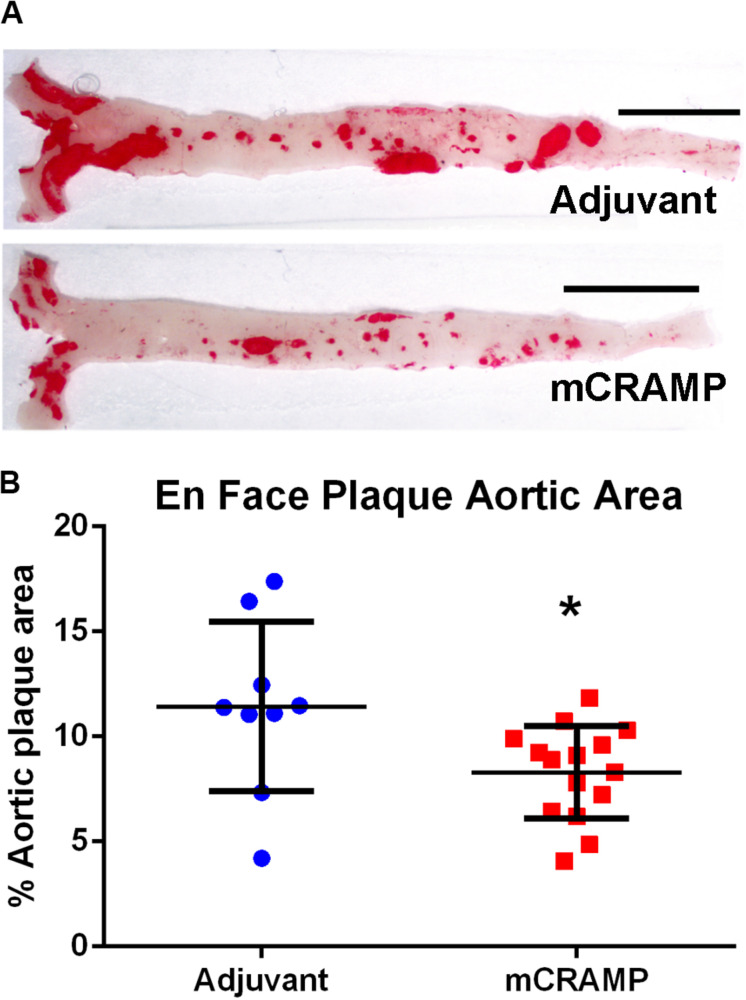
Aortic atherosclerosis plaque burden in T cell recipient apoE–/– mice. Representative photographs of en face aortas stained with Oil red O **(A)** from T cell recipients of adjuvant and mCRAMP immunized mice. Bar = 0.5 cm. Atherosclerotic burden was assessed by measuring Oil Red O stained plaque area **(B)**. Adjuvant *N* = 9; mCRAMP *N* = 15; *P < 0.05, *t*-test.

Recipients of T cells from mCRAMP immunized mice had significantly reduced aortic plaque area compared to recipients of T cells from adjuvant mice (28% reduction, *P* < 0.05, Figures 6A,B). There was no significant difference between groups in mean body weight (mCRAMP = 42 ± 6 gr; Adjuvant = 43 ± 6 gr) or mean serum cholesterol (mCRAMP = 1500 ± 294 mg/dL; Adjuvant = 1270 ± 366 mg/dL). Thus, atherosclerosis progression was reduced in the mCRAMP T cell recipient mice without differences in weight or serum cholesterol compared to adjuvant T cell recipient mice.

**FIGURE 7:**
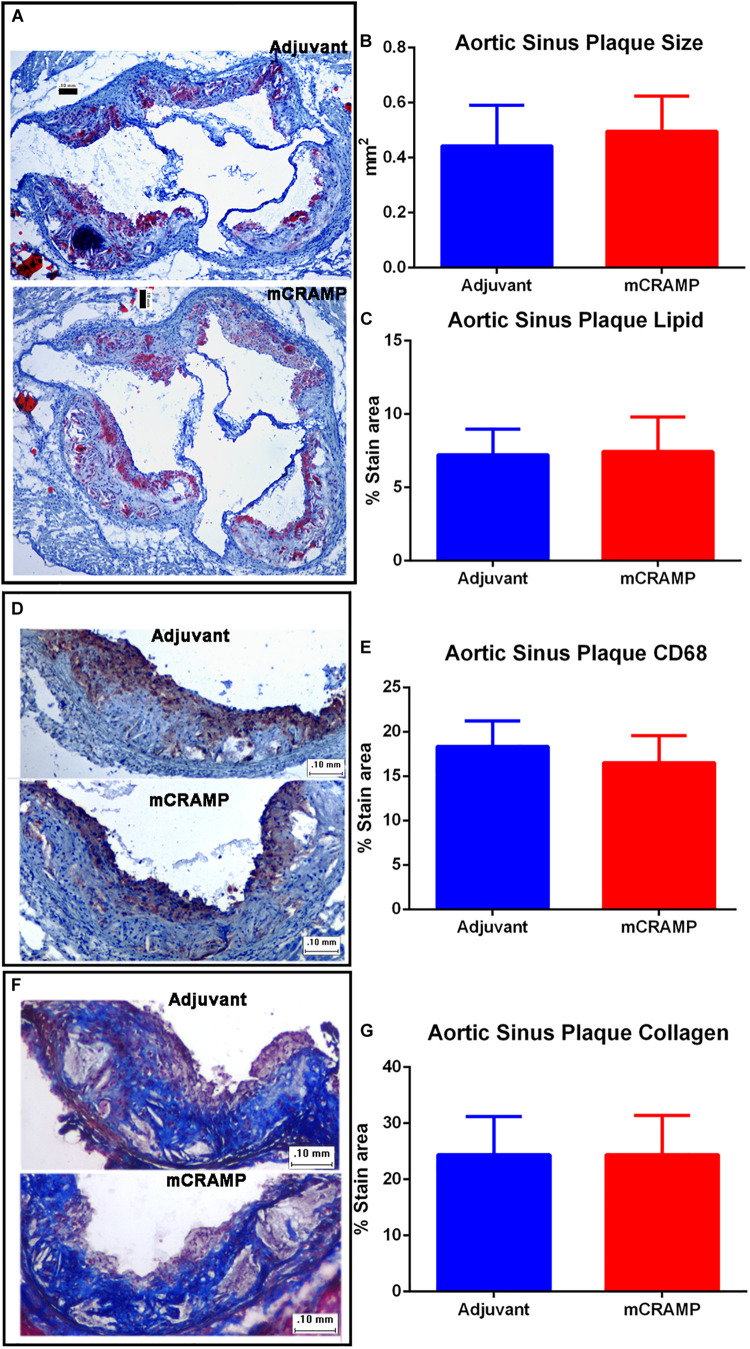
Aortic sinus plaque composition in T cell recipient apoE–/– mice. Representative photos of Oil Red O stain for lipids **(A)**. Aortic sinus plaque size (**B**; Adjuvant *N* = 9, mCRAMP *N* = 12) and lipid area (**C**; Oil Red O staining; Adjuvant *N* = 9; mCRAMP *N* = 12). Representative photos of CD68 stain for macrophage **(D)** and macrophage area (**E**; *N* = 6 each). Representative photos of Masson’s trichrome stain **(F)** and collagen stain area (**G**; Adjuvant *N* = 9; mCRAMP *N* = 15). Bar = 0.1 mm.

**FIGURE 8:**
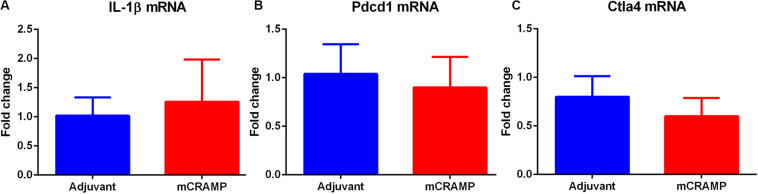
Analysis of key immune regulatory pathways in T cell recipient mice. Splenic mRNA expression of IL-1β **(A)**, Pdcd1 **(B)** or Ctla4 **(C)** between mCRAMP or adjuvant T cell recipient mice. *N* = 5 each.

Aortic sinus plaque size, lipid content (Oil Red O staining; Figures 7A–C), macrophage (CD68 staining; Figures 7D,E), and collagen area (Masson’s trichrome staining; Figures 7F,G) were not different between the T cell recipient groups. There were also no differences in IL-1β (Figure 8A), PDCD1 (Figure 8B) or cytotoxic T-lymphocyte-associated protein 4 (Ctla4, Figure 8C) splenic mRNA expression between the T cell recipient groups.

### Plaque Calcification in T Cell Recipient Mice

**FIGURE 9:**
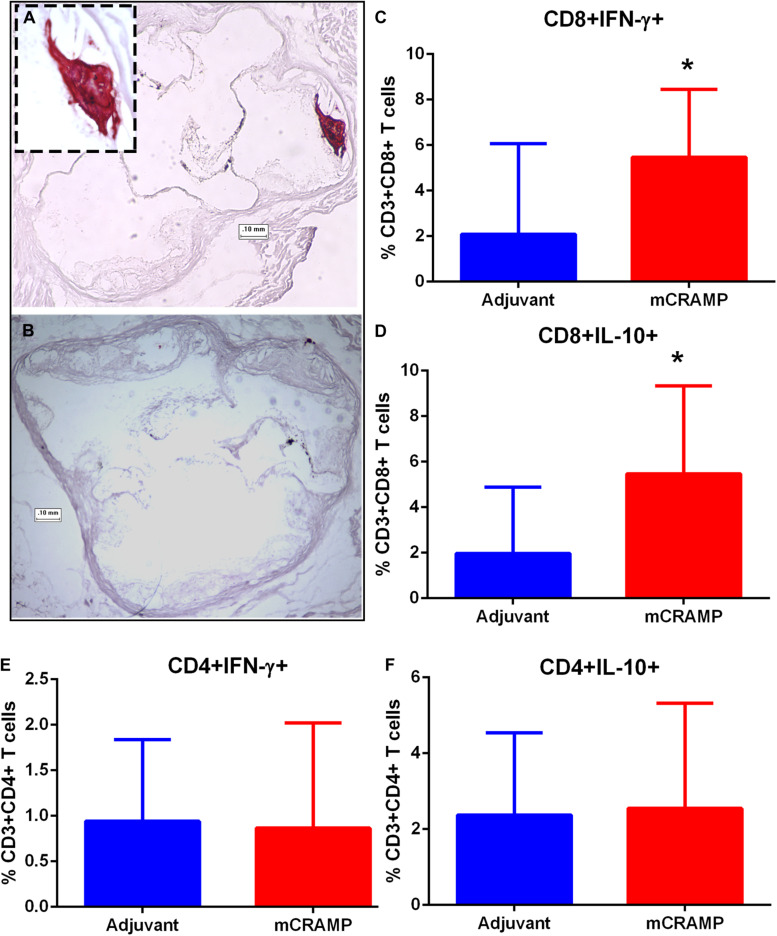
Atherosclerotic plaque calcification in T cell recipient mice. Representative photographs of aortic sinuses stained with the calcium specific Alizarin Red S in adjuvant (**A**, inset 20× magnification) or mCRAMP **(B)** T cell recipient mice. Bar = 0.1 mm. Intracellular staining for splenic CD8+INF-γ+ T cells **(C)** and CD8+IL-10+ T cells **(D)**. Intracellular staining for splenic CD4+INF-γ+ T cells **(E)** and CD4+IL-10+ T cells **(F)**. Adjuvant *N* = 8; mCRAMP *N* = 14. *P < 0.05, Mann-Whitney test. Gating scheme for T cell intracellular staining shown in Supplementary Figure 8.

Focal staining of hematoxylin was observed in several aortic sinus sections from recipient mice, suggesting calcification in the plaque. The presence of plaque calcification was assessed with the calcium-specific Alizarin Red S staining. Calcification in atherosclerotic plaques occurred in 56% of adjuvant T cell recipient control mice, compared to none in the mCRAMP T cell recipient mice (Figures 9A,B and Table 2; Fisher’s exact test *P* = 0.003).

**TABLE 2:** Fisher’s Exact test of aortic sinus plaque calcification prevalence in T cell recipient mice (two-sided *P* = 0.003).

	Number of mice with plaque calcification	Number of mice without plaque calcification	Total
Adjuvant	5	4	9
mCRAMP	0	15	15
Total	5	19	24

**FIGURE 10:**
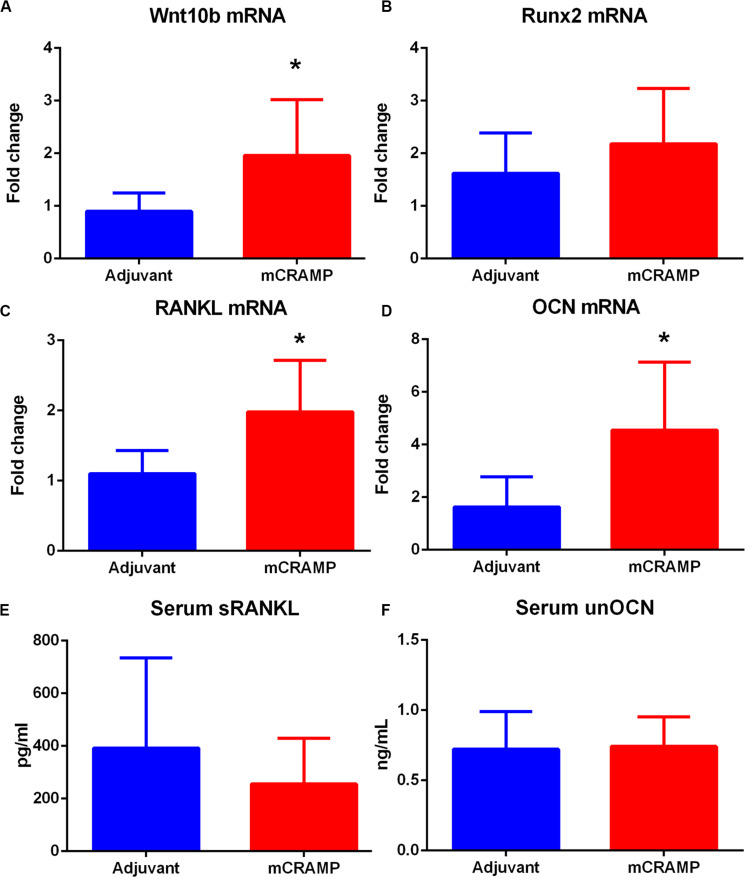
Pathways of tissue calcification potentially mediated by T cells. **(A)** Splenic Wnt10b mRNA expression in mCRAMP compared to adjuvant T cell recipient mice at 23 weeks of age (**A**; *N* = 7 each group). Splenic Runx2 mRNA (**B**; *N* = 5 each group), RANKL mRNA **(C)**, and osteocalcin (OCN) mRNA **(D)** expression in mCRAMP T cell recipient mice compared to adjuvant T cell recipients (*N* = 5–7 each). Serum RANKL (**E**; Adjuvant *N* = 9; mCRAMP *N* = 14) and serum undercarboxylated osteocalcin (unOCN) levels (*F*; Adjuvant *N* = 9; mCRAMP *N* = 14). *P < 0.05; **(A,C)**, *t*-test; **(D)**, Mann-Whitney test.

Immune pathways associated with T cell mediated tissue calcification were then investigated further. Recipients of T cells from mCRAMP immunized mice had increased IL-10 and IFN-γ expression in splenic CD8+ T cells (Supplementary Figure 8 and Figures 9C,D), but not in CD4+ T cells (Figures 9E,F), compared to adjuvant T cell recipient control mice. There was increased expression of Wnt10b mRNA in splenocytes of mCRAMP T cell recipients, when compared to adjuvant T cell recipient control mice (Figure 10A). However, there was no difference in the expression of Runx2 mRNA (Figure 10B). Furthermore, splenocytes from mCRAMP T cell recipients had increased RANKL and osteocalcin mRNA expression when compared to adjuvant (Figures 10C,D). However, no difference between groups was noted in serum RANKL and undercarboxylated osteocalcin concentration (Figures 10E,F), which is the known active state of osteocalcin.

## Discussion

In this study, we showed that: (a) LL-37 stimulation of PBMCs from patients with ACS induced the persistence of CD8+ TEM cell response compared to patients with stable CAD or self-reported controls; (b) Immunization of apoE−/− mice with mCRAMP, the cationic fragment of CRAMP, increased CD8 CM T cell activation and cytolytic activity; and (c) Adoptive transfer of T cells from mice immunized with mCRAMP was associated with smaller atherosclerotic aortic plaque area, and absence of aortic sinus plaque calcification.

Our findings suggest that LL-37 self-reactive T cells may be important in an acute coronary event in the context of atherosclerotic disease. LL-37 is a T cell self-antigen in patients with psoriasis (15), who have increased risk of early cardiovascular disease (16, 17). It has been proposed that psoriasis and atherosclerosis, both chronic inflammatory conditions, share a common inflammatory pathogenic basis (21, 22) that can potentially explain the increased risk of atherosclerosis and its complications associated with this condition. TEM cells have rapid effector function upon re-exposure to the antigen, but a limited proliferative potential (23). Memory T cells correlate well with atherosclerosis in both mice and humans (5, 24), and with vulnerable and ruptured plaques in humans (6).

LL-37 was reported to decrease T cell proliferation in resting human PBMCs with increased cell viability without changes in CD4+FoxP3+ *T*_reg_ cell percentage suggesting that in the steady-state, LL-37 treatment results in a degree of immune-modulation apparently independent of increased *T*_regs_ (25). In the same report, T cells increased proliferation when co-activated with phytohaemagglutinin without affecting cell viability and with increased *T*_reg_ cells. Increased T cell proliferation coupled with increased *T*_reg_ cells in their report suggests that *T*_regs_ may be compensating for the increased proliferation, or that *T*_regs_ may not have a significant role in LL-37 reactive T cells. Our findings extend their report by demonstrating that T cell memory response to LL-37 was reduced in the control subjects and stable CAD but persisted in samples from ACS patients. This is supported by the qualitative difference in the IFN-γ secretion among the groups. These differences were associated with reduced immune checkpoint PDCD1 mRNA expression in PBMCs from ACS patients. The reduction in T cell response to LL-37 in the control PBMC was blocked by HLA Class-I antibody suggesting that the intrinsic response is at least partially MHC-I dependent. However, our results cannot rule out the possibility that adjuvant effects attributed to LL-37 are also involved (15). The observed blocking of complementary CD4+ T cell memory response by anti-HLA Class-I antibody is consistent with the report by Lande et al. (15) suggesting complementarity of T cell subset responses to LL-37. Whether the complementary T cell response to LL-37 in the controls also extend to ACS as was reported for some psoriasis patients (15) remains to be determined. Thus, combined with other reports, our results suggest that the intrinsic T cell response to LL-37 is down-modulation but in the presence of co-activating factors such as those in the ACS patients the T cell memory response persists that may be due in part to reduced checkpoint PDCD1 expression. These findings have potentially important clinical implications with the reported cases of ACS associated with immune checkpoint inhibitor treatment (26).

The results in our report may be a response to the acute event but adaptive immune memory to neoantigens develop over a longer time frame than that in our study (within 72 h of admission for the acute event). It is notable that CD8+ TEM response to LL-37 is consistent in patients whether it was their first ACS event or a recurrent event suggesting the continued presence of memory T cells. It cannot be determined at this time whether the persistent T cell response in ACS is pathogenic or a compensatory response in the acute stage of the disease. On the other hand, the CD4+ T Effector response was altered in patients that had a recurrent event suggesting that the T cell response in ACS evolved as the recurrent patients remained at risk. These observations are consistent with the notion of underlying inflammation in patients who remain at risk for a recurrent event.

Our results further show a correlation between the CD4+ T cell response to LL-37 and cat-hCAP-18. This finding may be a manifestation of expanding antigenic determinants reported in antigen spreading, wherein the original reactive antigen determinant spreads to other regions of the same protein (27), in this case hCAP-18. Although speculative, it is interesting that this was not observed in CD8+ T cell response. It remains to be determined what the nature of the involvement of the TEM response to LL-37 is in the acute event.

Studies were performed in apoE−/− mice to investigate the role of T cells reactive to the cationic antimicrobial peptide in atherosclerosis. Although the cathelin domain of the proprotein hCAP-18/CRAMP is reported to be active (28), functional activity is mostly attributed to the cationic antimicrobial peptide domain. Our results show increased CD8+ CM T cells and CD8+ T cell cytotoxic activity in mice immunized with the self-peptide mCRAMP coupled with decreased CD8+FoxP3+ T cells. These extend our previous report where immunization with the proprotein CRAMP resulted in increased CD8+ T cells and cytolytic activity, and reduced atherosclerosis (19). CD8+ *T*_regs_ have an important role in self-tolerance, and lower levels CD8+ *T*_regs_ have been associated with increased immune activity (29–31).

We confirmed that T cells reactive to mCRAMP are functionally involved in atherosclerosis by the adoptive transfer of enriched T cells from mCRAMP-primed apoE−/− mice into recipient apoE−/− mice. To the best of our knowledge, this is the first report of reduced atherosclerosis attributed to T cells primed with mCRAMP. However, no differences were found in aortic sinus plaques consistent with the report of site-specificity for atherosclerosis in murine models of atherosclerosis (32).

Some key signaling pathways involved in tissue calcification potentially regulated by T cells in the recipient mice were investigated. Wnt10b, a member of the Wnt family signaling pathway, is secreted by T lymphocytes (33, 34) and activates signal transduction cascades that regulate Runx2, a transcription factor needed for osteoblast differentiation (35). RANKL activates osteoclasts and bone remodeling in adult mice (36). T cell expression of RANKL (37) is involved in the regulation of bone metabolism (38). Osteocalcin is secreted by osteoblasts associated with bone formation. Splenocytes expressing osteocalcin induce atherosclerosis and vascular calcification in apoE−/− mice (39). Although the dysregulation of calcification pathways is manifested in differential mRNA expression of specific genes involved, the systemic levels detected in serum seemed to remain in equilibrium. This is further observed in the increase in both IL-10 and IFN-γ expressing CD8+ T cells. The results suggest the involvement of factors that remain to be characterized.

Nevertheless, these observations suggest dysregulated tissue calcification pathways in atherosclerosis potentially mediated by memory T cells. Interestingly, LL-37 is a T cell antigen in psoriatic disease (15) and altered bone remodeling through osteoblast–osteoclast uncoupling has been proposed as an explanation for the concomitant dysregulated processes of both pathological bone formation and resorption usually found in patients with psoriatic arthritis (40). In our study, plaque calcification was absent in mice that were recipient of T cells primed with mCRAMP suggesting a role in regulating the process.

Coronary artery calcification is a marker of atherosclerosis and its role in CAD is nuanced (41). On one hand, the widely used coronary artery calcium score is a strong independent risk factor of major adverse cardiovascular and cerebrovascular events (42, 43). On the other hand, calcified atherosclerotic plaques may be more stable than non-calcified plaques (44). The use of statins has been associated with progression of plaque calcification, in spite of their protective role in atherosclerosis (45). The association found in our animal experiments between recipients of mCRAMP-primed T cells and the lack of plaque calcification is intriguing, but its significance remains to be determined. The role of T cells reactive to LL-37 in humans, and their potential influence in the pathways of atherosclerotic plaque calcification need to be investigated further.

Limitations of the study include the potential of LL-37 to be a “promiscuous” HLA-binding peptide or to have intrinsic adjuvant properties (15) which coupled with the reduced PDCD1 expression in ACS may explain the observed persistence of CD8+ Effector cells. Although anti-HLA Class-I antibody blocked the response to LL-37 in controls, our study cannot completely exclude these possibilities. The mouse studies demonstrating the involvement of T cells reactive to mCRAMP as a self-antigen in atherosclerosis is consistent with the observed TEM cell response to LL-37 in ACS patients, supporting the presence of a self-reactive TEM cell population involved in atherosclerosis. However, the persistence of TEM response to LL-37 in patients who suffered an acute event is not in complete alignment with the results of adoptive transfer of mCRAMP-primed T cells in our mouse studies. One might speculate that the controlled nature of immune priming in the mice skewed the response to be protective against atherosclerosis, even as the physiologic role of reduced calcification of mouse plaques remains to be clarified. It is also possible that the persistence of LL-37 reactive TEM response in ACS patients is a compensatory response to the inflammatory milieu that subsequently proved inadequate. These remain speculative handicapped by several limitations, including the lack of a reliable mouse model of spontaneous coronary artery plaque rupture which is the major cause of ACS in humans.

## Data Availability Statement

The original contributions presented in the study are included in the article/Supplementary Material, further inquiries can be directed to the corresponding author.

## Ethics Statement

The studies involving human participants were reviewed and approved by Cedars-Sinai IRB. The patients/participants provided their written informed consent to participate in this study. The animal study was reviewed and approved by Cedars-Sinai Institutional Animal Care and Use Committee.

## Author Contributions

FC, BC, WL, PM, and PD contributed to conception and design of the study. FC, WL, JY, PM, XZ, JZ, and PD contributed to the data acquisition and analysis. BC and RH contributed to patient recruitment. FC, BC, WL, K-YC, PS, and PD contributed to the interpretation of the data, drafting, and revising the manuscript. All authors contributed to the article and approved the submitted version.

## Conflict of Interest

The authors declare that the research was conducted in the absence of any commercial or financial relationships that could be construed as a potential conflict of interest.
