# Supplementary material for: The Role of T Cells Reactive to the Cathelicidin Antimicrobial Peptide LL-37 in Acute Coronary Syndrome and Plaque Calcification
Source: Front Immunol. 2020 Oct 6;11:575577. doi: 10.3389/fimmu.2020.575577 (PMC7573569; doi:10.3389/fimmu.2020.575577)
Supplement: Supplementary file 1 [file Data_Sheet_1.PDF]

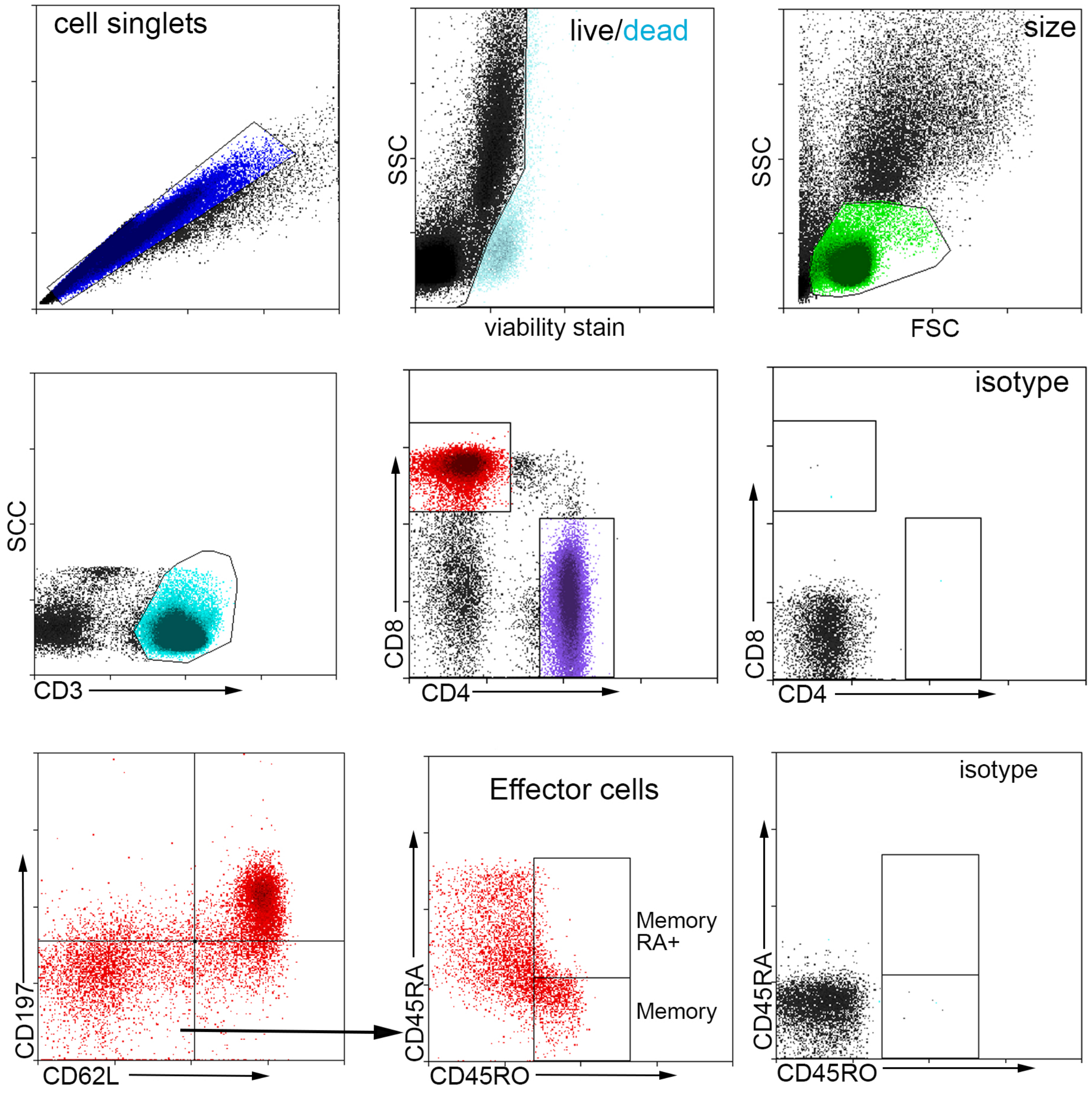

Supplementary Figure 1: Gating scheme for hPBMCs. Human PBMCs were stimulated with either cat-hCAP-18 or LL-37 for 72 hours. No peptide stimulation and cell stimulation cocktail treatment served as negative and positive controls, respectively. Cell singlets were size gated after exclusion of non-viable cells. CD4+ or CD8+ T cells were selected from CD3+ T cells. CD197(-)CD62L(-) cells were further selected for plotting on CD45RO and CD45RA. Isotypes served as reference.
