# Supplementary material for: The Role of T Cells Reactive to the Cathelicidin Antimicrobial Peptide LL-37 in Acute Coronary Syndrome and Plaque Calcification
Source: Front Immunol. 2020 Oct 6;11:575577. doi: 10.3389/fimmu.2020.575577 (PMC7573569; doi:10.3389/fimmu.2020.575577)
Supplement: Supplementary file 2 [file Data_Sheet_2.PDF]

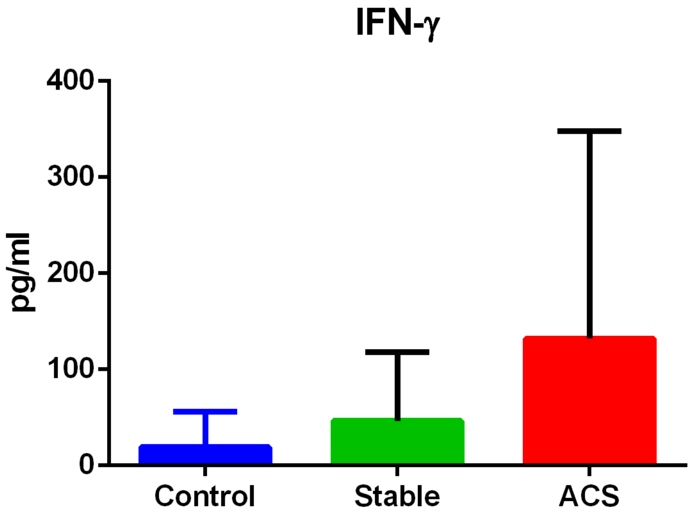

Supplementary Figure 2: IFN-γ in conditioned medium of LL-37 stimulated peripheral blood mononuclear cells from control subjects and patients. Peripheral blood mononuclear cells were treated with LL-37 for 72 hours. Medium was added at 1/3 original volume to replenish nutrients at the 48h time-point. Conditioned medium was collected and ELISA for IFN-γ was performed. Control N=7; Stable N=5; ACS N=9.
