# Supplementary material for: The Role of T Cells Reactive to the Cathelicidin Antimicrobial Peptide LL-37 in Acute Coronary Syndrome and Plaque Calcification
Source: Front Immunol. 2020 Oct 6;11:575577. doi: 10.3389/fimmu.2020.575577 (PMC7573569; doi:10.3389/fimmu.2020.575577)
Supplement: Supplementary file 3 [file Data_Sheet_3.PDF]

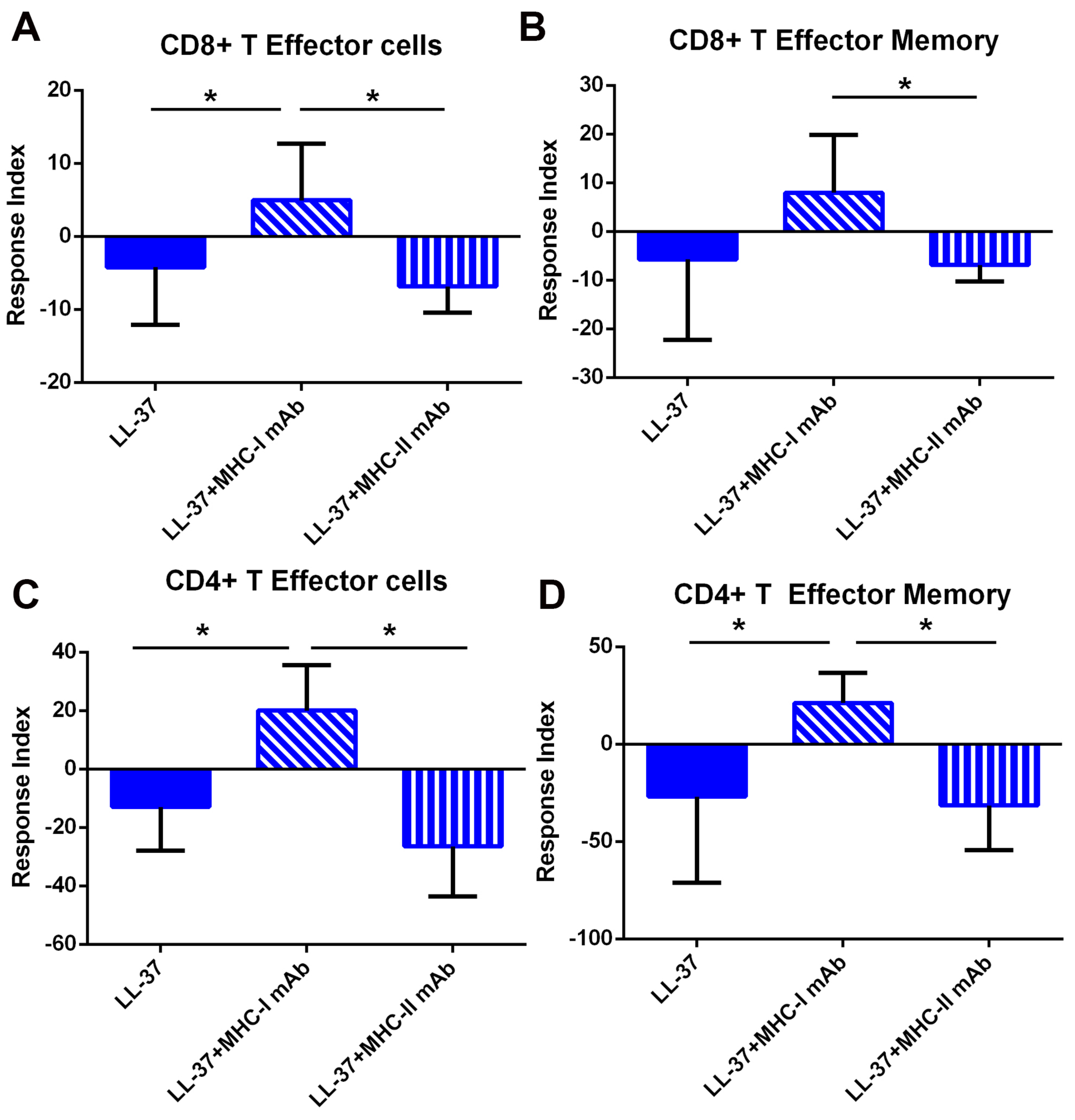

Supplementary Figure 3: Anti HLA Class-I and anti HLA Class-II antibody blocking. Peripheral blood mononuclear cells from control subjects were stimulated with LL-37 in the presence or absence of blocking antibodies against HLA Class-I (MHC-I mAb) or HLA Class-II (MHC-II mAb). N=8-9 each; A and C ANOVA and Holm-Sidak test; B and D Kruskal-Wallis and Dunn’s test; *$P<0.05$.
