# Supplementary material for: The Role of T Cells Reactive to the Cathelicidin Antimicrobial Peptide LL-37 in Acute Coronary Syndrome and Plaque Calcification
Source: Front Immunol. 2020 Oct 6;11:575577. doi: 10.3389/fimmu.2020.575577 (PMC7573569; doi:10.3389/fimmu.2020.575577)
Supplement: Supplementary file 4 [file Data_Sheet_4.PDF]

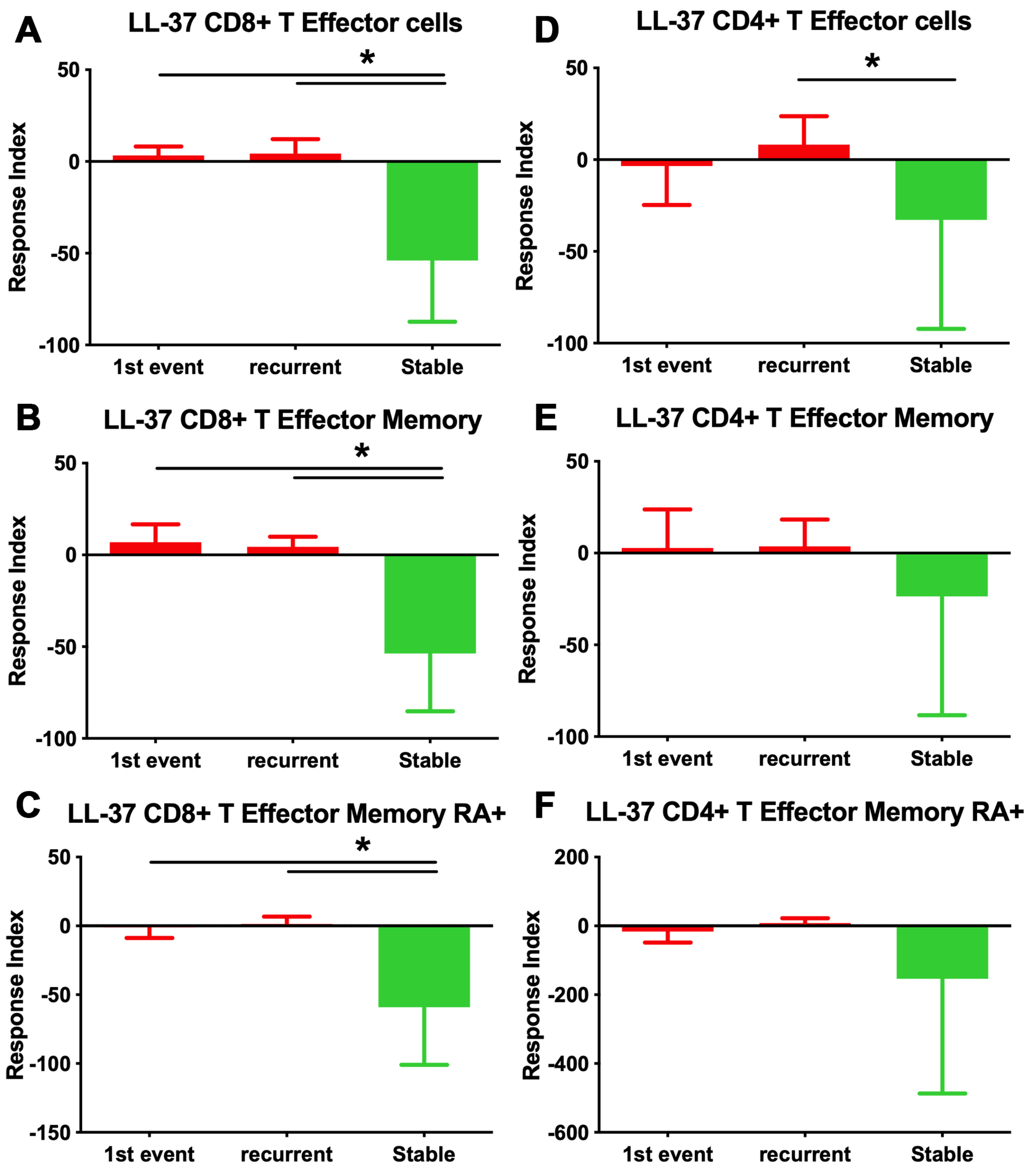

Supplementary Figure 4: T cell response to LL-37 in patients with first ACS or recurrent ACS. CD8+ (A-C) and CD4+ (D-F) Memory T cell responses to LL-37 stimulation of peripheral blood mononuclear cells from patients reported with their first acute coronary syndrome (ACS) event (1st event) or a recurrent ACS event (recurrent) compared to stable coronary artery disease (Stable) patients. 1st event N=6; recurrent N=4; Stable N=10; *P < 0.05.
