# Supplementary material for: The Role of T Cells Reactive to the Cathelicidin Antimicrobial Peptide LL-37 in Acute Coronary Syndrome and Plaque Calcification
Source: Front Immunol. 2020 Oct 6;11:575577. doi: 10.3389/fimmu.2020.575577 (PMC7573569; doi:10.3389/fimmu.2020.575577)
Supplement: Supplementary file 6 [file Data_Sheet_6.PDF]

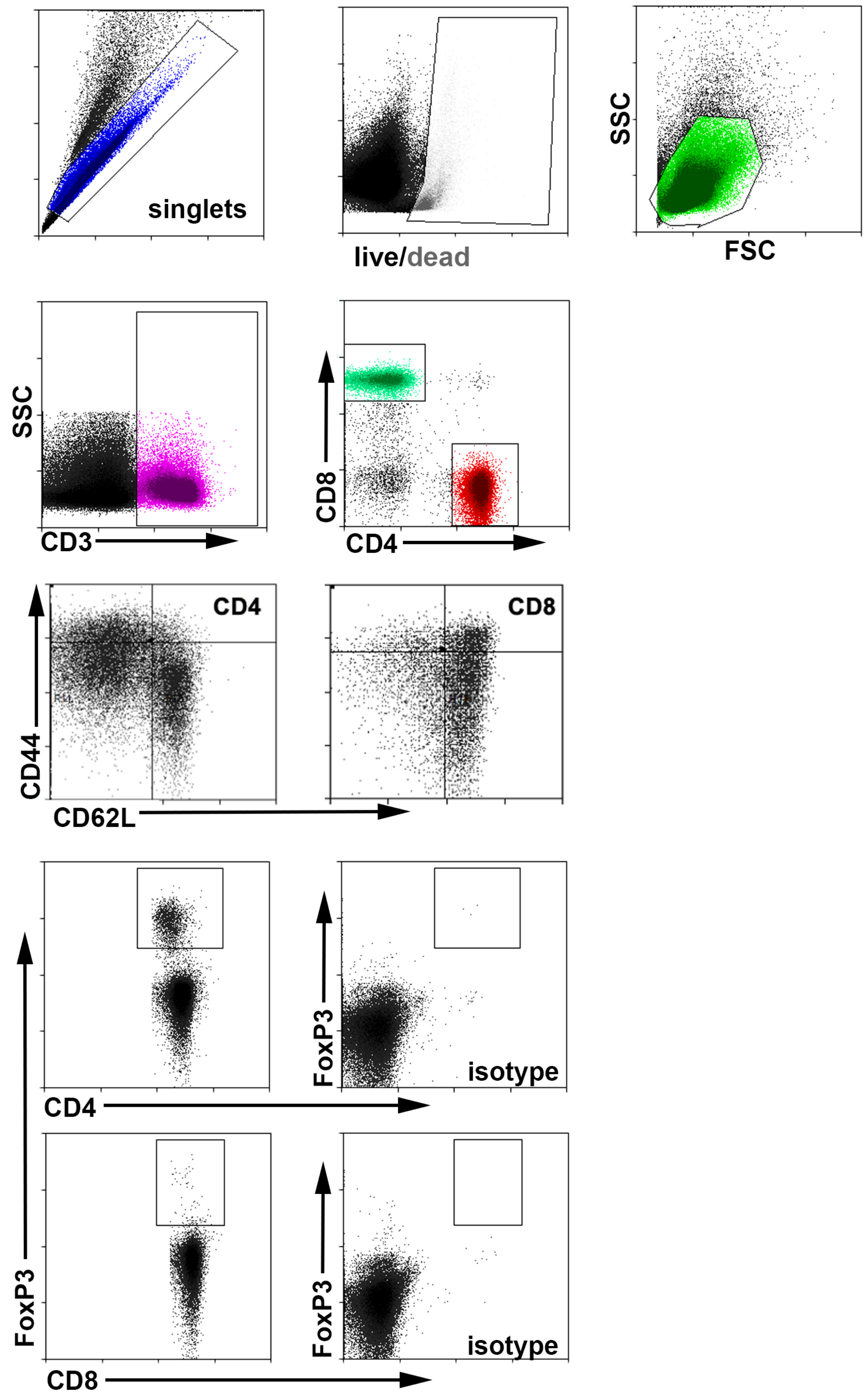

Supplementary Figure 6: Gating scheme for memory T cells and FoxP3+ T cells. Splenocytes were selected for singlet cells, and size gated after exlcuding non-viable cells. CD3+ T cells were selected for CD4+ or CD8+ T cells and plotted on CD44 and CD62L for Memory T cells. An aliquot of cells were processed for intracellular staining for FoxP3+ T cells.
