# Supplementary material for: The Role of T Cells Reactive to the Cathelicidin Antimicrobial Peptide LL-37 in Acute Coronary Syndrome and Plaque Calcification
Source: Front Immunol. 2020 Oct 6;11:575577. doi: 10.3389/fimmu.2020.575577 (PMC7573569; doi:10.3389/fimmu.2020.575577)
Supplement: Supplementary file 7 [file Data_Sheet_7.PDF]

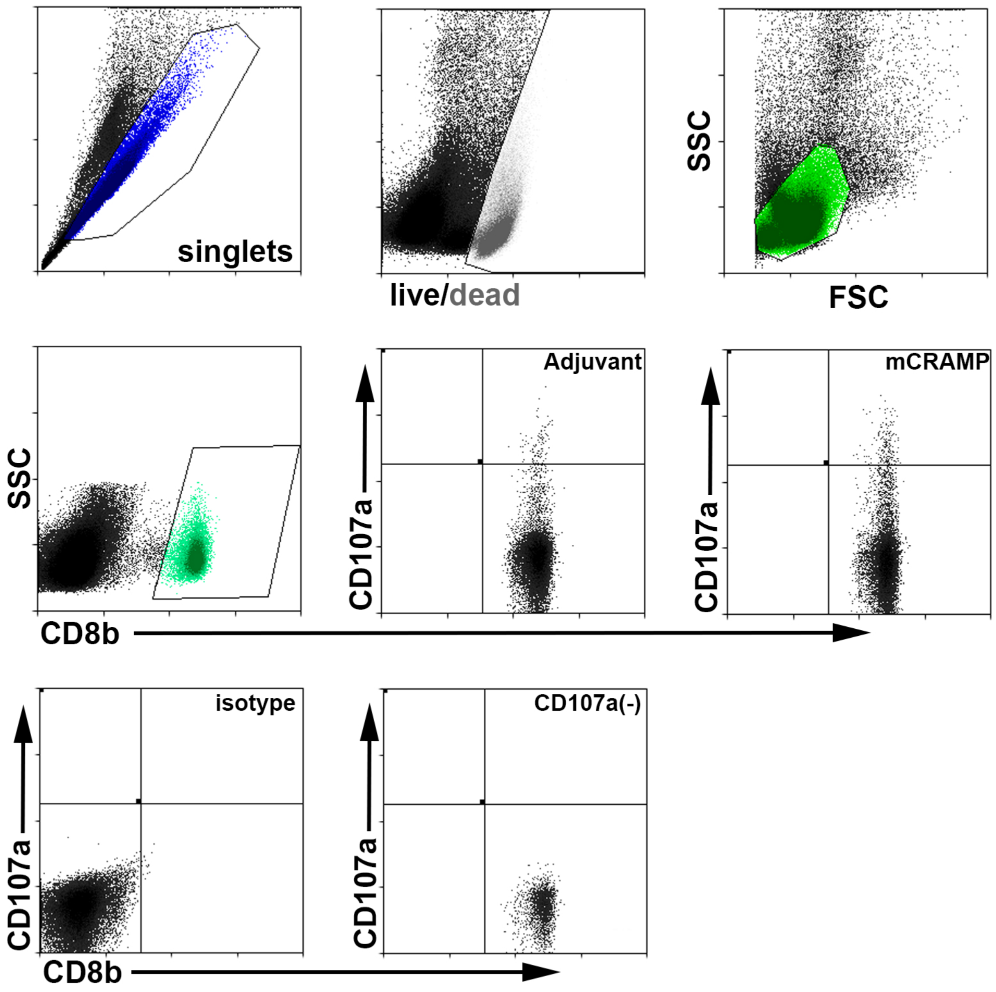

Supplementary Figure 7: Gating scheme for CD8+CD107a+ T cells. Splenocytes were gated for cell singlets and non-viable cells excluded. Size-gated cells were selected for CD8b and plotted on CD107a. Representative scatter plots of CD8+CD107a+T cells from Adjuvant and mCRAMP immunized mice shown. Isotype and CD8-gated staining controls were used as reference for analysis.
