# Supplementary material for: The Role of T Cells Reactive to the Cathelicidin Antimicrobial Peptide LL-37 in Acute Coronary Syndrome and Plaque Calcification
Source: Front Immunol. 2020 Oct 6;11:575577. doi: 10.3389/fimmu.2020.575577 (PMC7573569; doi:10.3389/fimmu.2020.575577)
Supplement: Supplementary file 8 [file Data_Sheet_8.PDF]

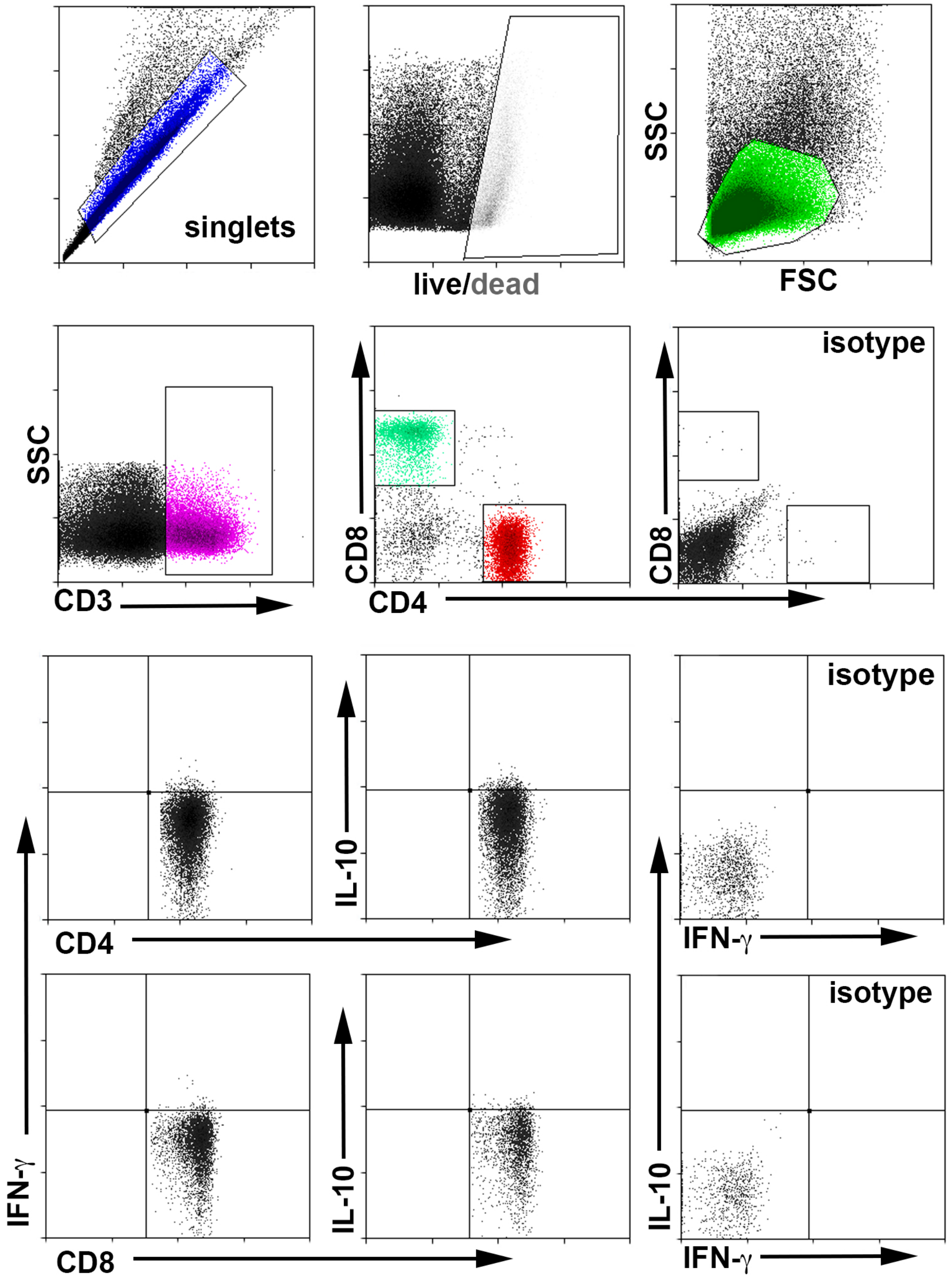

Supplementary Figure 8: Gating scheme for intracellular cytokine staining. Splenocytes were incubated in 1x Monensin for 4 hours and processed for intracellular staining. Cell singlets excluding non-viable cells were selected for size and CD3+ T cells selected for CD4 or CD8 expression. CD4+ or CD8+ T cells were then plotted on IFN-γ or IL-10. Isotypes were used for reference.
